# Enhanced ResNet-50 with Multi-Feature Fusion for Robust Detection of Pneumonia in Chest X-Ray Images

**DOI:** 10.3390/diagnostics15162041

**Published:** 2025-08-14

**Authors:** Neenu Sebastian, B. Ankayarkanni

**Affiliations:** Department of Computer Science and Engineering, School of Computing, Sathyabama Institute of Science and Technology (Deemed to be University), Chennai 600119, India

**Keywords:** attention mechanism, chest X-ray, deep learning, feature fusion, pneumonia detection, texture features, ResNet-50

## Abstract

**Background/Objectives:** Pneumonia is a critical lung infection that demands timely and precise diagnosis, particularly during the evaluation of chest X-ray images. Deep learning is widely used for pneumonia detection but faces challenges such as poor denoising, limited feature diversity, low interpretability, and class imbalance issues. This study aims to develop an optimized ResNet-50 based framework for accurate pneumonia detection. **Methods:** The proposed approach integrates Multiscale Curvelet Filtering with Directional Denoising (MCF-DD) as a preprocessing step to suppress noise while preserving diagnostic details. Multi-feature fusion is performed by combining deep features extracted from ResNet-50 with handcrafted texture descriptors such as Local Binary Patterns (LBPs), leveraging both semantic and structural information. Precision attention mechanisms are incorporated to enhance interpretability by highlighting diagnostically relevant regions. **Results:** Validation on the Kaggle chest radiograph dataset demonstrates that the proposed model achieves higher accuracy, sensitivity, specificity, and other performance metrics compared to existing methods. The inclusion of MCF-DD preprocessing, multi-feature fusion, and precision attention contributes to improved robustness and diagnostic reliability. **Conclusions:** The optimized ResNet-50 framework, enhanced by noise suppression, multi-feature fusion, and attention mechanisms, offers a more accurate and interpretable solution for pneumonia detection from chest X-ray images, addressing key challenges in existing deep learning approaches.

## 1. Introduction

Pneumonia is a critical respiratory illness that predominantly affects the tiny air sacs within the lungs that are essential for gas exchange. During infection, these sacs become filled with fluid, leading to breathing difficulties. Morbidity and mortality from pneumonia remain high, especially in populations with preexisting health conditions [1].

Chest X-rays are widely utilized for pneumonia diagnosis due to their rapid imaging capabilities and cost efficiency [2]. However, the visual patterns associated with pneumonia infection can overlap with other lung pathologies, making accurate diagnosis challenging for radiologists. The use of AI in developing automated solutions for pneumonia diagnosis has grown substantially. Manual feature extraction was a prerequisite in traditional machine learning frameworks like supervised learning [3], where these features served as inputs to the classification algorithms. On the other hand, deep learning models [4,5] are capable of learning by extracting the features by themselves without any need of manual interference.

Though CNN has been preferred as the current standard practice for medical image analysis, it has several challenges in the same field. Existing CNN models inadequately handle image noise, often applying generic denoising techniques that either fail to suppress artifacts or compromise clinically relevant structures. CNN has greater reliance on deep features, which results in the neglection of fine-grained texture patterns. These texture patterns like pixel intensity, edge variations, etc., are vital pneumonia infection signs. The interpretability of such models also remains limited, with few offering visual explanations or attention maps that can support clinical decision making. Standard pneumonia detection datasets have imbalanced class distributions, which results in biased model learning and reduced generalization. In response to these issues, efforts have been directed toward building a resilient automated model for detecting pneumonia.

A hybrid deep learning model that effectively merges deep semantic features with fine-grained handcrafted descriptors is described in this work to solve the above said challenges. The proposed framework employs the Multiscale Curvelet Filtering with Directional Denoising (MCF-DD) method to effectively suppress noise in the input images. By integrating attention modules, the system directs its focus toward pneumonia infected areas, thereby boosting diagnostic accuracy and enhancing the model’s interpretability.

The primary contributions of this study are summarized as follows:

- Enhanced ResNet-50-Based Architecture: A customized ResNet-50 model was adapted and fine tuned for grayscale chest X-ray images to facilitate faster training convergence and enhance feature extraction specific to medical imaging.
- Noise-Robust Preprocessing with MCF-DD: For better diagnostic accuracy in chest radiographs, the proposed framework integrates Multiscale Curvelet Filtering with Directional Denoising (MCF-DD) as a preprocessing step. This technique dynamically identifies and suppresses both Poisson and Gaussian noise while preserving fine structural and anatomical details critical for accurate diagnosis.
- Hybrid Feature Fusion (HFF): A hybrid fusion strategy of deep multiscale features from ResNet-50 with handcrafted descriptors such as local binary patterns (LBPs) is introduced, improving feature diversity and enhancing pneumonia classification.
- Incorporation of Attention Mechanism: Incorporating CBAM allowed the model to selectively attend to spatial and channel-wise informative features, particularly those associated with pneumonia lesions, leading to improved diagnostic accuracy and model transparency.

## 2. Related Works

Pneumonia infection is one of the factors contributing to the higher mortality of young children less than 5 years and adults with low immunity. The high mortality rate due to COVID-19 infection which caused pneumonia is one incident which gives a clear picture of the extent to which it can affect human life.

**Figure 1:**
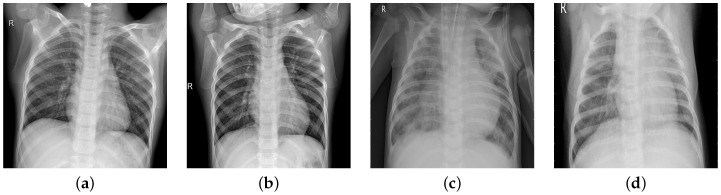
Normal images (**a**,**b**); pneumonia images (**c**,**d**).

In chest radiographs radiographic features of a normal person include clear dark lung fields, well-defined lung borders, clear vascular markings, and a midline trachea. The chest radiograph of a person infected with pneumonia will exhibit localized or diffuse opacities, caused by fluid or pus-filled alveolar spaces. In Figure 1, we can observe the differences in the two types of images.

The recent studies on pneumonia detection systems are based on CNN-based deep learning algorithms due to their remarkable results. Current models for pneumonia detection can be broadly classified into machine learning techniques, deep learning frameworks, transfer learning models, and ensemble approaches.

In machine learning (ML) approaches, the features are extracted manually and these features are given as input to machine learning classification models [6,7,8]. Transparency, low computational needs, and lesser resources were some of the advantages of machine learning approaches. But to identify the complex visual patterns present in chest X-rays, it is necessary to have strong feature representation. Recently, hybrid methods have been developed by combining deep features from CNNs with classical ML classifiers, resulting in improved performance [9,10,11]. The major challenges of this approach are scalability and feature robustness. Numerous research studies [12,13] have proved the effectiveness of deep learning approaches. Their ability to model a large number of spatial and textural features helped to perform the classification accurately. Unlike machine learning models, CNN has the ability to capture complex and significant features using backpropagation [14,15,16]. In a recent study [17], the robustness of CNN models to different imaging conditions is highlighted, which is critical for the accurate diagnosis of the disease in remote areas where medical group support is less available. Performing the training on smaller datasets can lead to problems, like overfitting, poor generalization, etc. Also, incorrect diagnosis can occur as a result of poorly labeled datasets. Several customized CNN architecture designs [18,19,20] are specifically tailored for pneumonia detection. These architectures contain custom layers, specialized filters, or specific configurations to optimize performance for tasks for pneumonia detection in medical images. Some popular CNN architectures which can be used for pneumonia classification are ResNet, DenseNet, VGG, and Inception.

Even though CNN has the ability to extract meaningful features from images, it requires large labeled data for training. To solve this drawback of CNN, transfer learning approaches were used, where the pretrained CNN models solve unseen new problems. In [21], five ResNet variants were used for pneumonia detection in chest rays and the customized ResNet model achieved higher accuracy. In [22], VGG 19, Xception, ResNet-50, and DenseNet121 CNN architectures were used to classify pediatric chest rays for pneumonia detection and Xception gave better results. Several other works [23,24,25,26,27,28] also applied transfer learning for pneumonia classification. The strengths of transfer learning approaches are the need for less labeled data and less training time. The application of transfer learning models leads to overfitting as the training is performed on homogeneous data, which can lead to strong performance on training data and poor generalization on unseen cases.

Later, ensemble learning methods were developed where multiple model predictions are combined to generate a better generalized prediction. The different ensemble learning approaches can be categorized by bagging, boosting, and stacking methods. Bagging involves training several instances of the same model independently, each on a different bootstrap sample drawn from the original training data. In [29], MobileNetV2 and NASNetMobile were used together with a weighted average bagging scheme, which achieved 98.63% accuracy while maintaining computational efficiency suitable for a low-resource setting. Classifiers are used together using the simple bagging method like voting in [30], which demonstrated strong recall of 95.6% for classifications. In boosting, training of the models is performed sequentially, where each successive model is trained specifically to address and reduce cumulative prediction error by learning from the mistakes of prior models. The weighted sum of the predictions from all the models is calculated as the final prediction and more weight is given to models with better performance. In stacking, base learners are trained and then meta learner is used to combine their predictions. Some recent works using stack approaches are [31,32,33,34,35,36]. The challenges of ensemble learning are high computational cost and increased model complexity. Recent studies on pneumonia detection from chest X-ray images are summarized in Table 1.

Even though there are significant advantages to applying AI to pneumonia detection in chest X-rays, existing approaches exhibit notable limitations. The dependency on handcrafted features, which lack the capability of capturing radiographic patterns and poor generalization, is a challenge faced by machine learning approaches. On the other hand, deep learning approaches are powerful, but the working of deep learning approaches is like black boxes. Also, the demand for large labeled datasets raises challenges in medical image environments. The need of training time is reduced in transfer learning, but if the source and targets are of different domains, it can lead to poor domain adaptation. Ensemble models improve accuracy, but it causes complexity, redundancy, and reduced interpretability.

To address these gaps, our study proposes an enhanced ResNet-50 based architecture, in which the input images are preprocessed using a Multiscale Curvelet Filtering with Directional Denoising framework to improve image quality by suppressing noise and enhancing edge details. The handcrafted texture descriptors with deep features are fused together to enhance the representation capacity. The class imbalance problem is addressed by using the weighted random sampler function. Overall, our approach presents a balanced, interpretable, and computationally efficient solution without the complexity of traditional ensemble techniques.

## 3. Methodology

**Figure 2:**
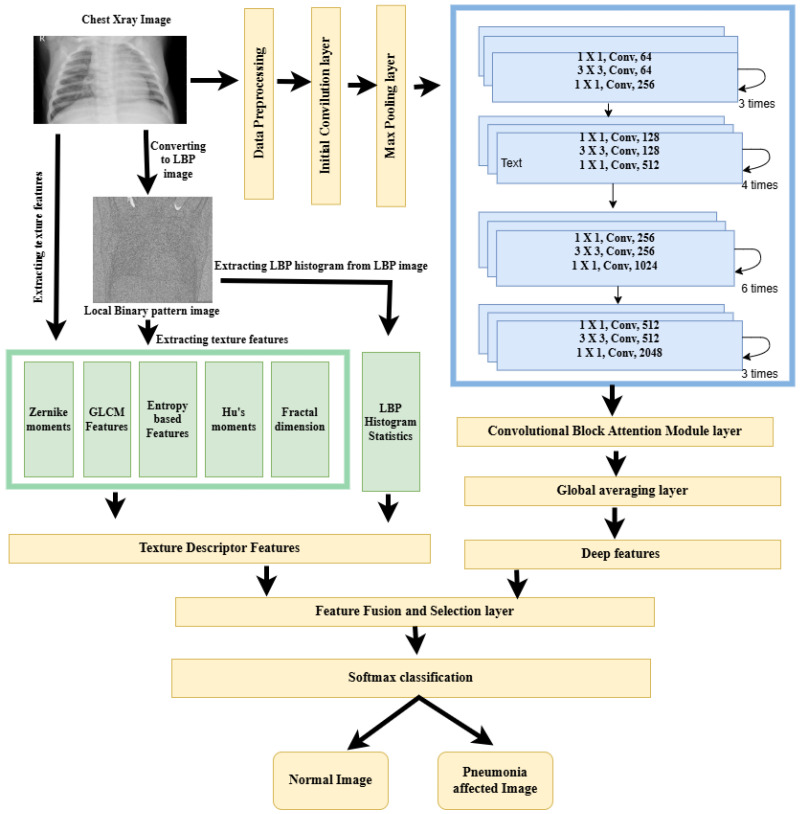
Enhanced ResNet-50 framework.

The proposed framework uses standard ResNet-50 as the backbone structure. Initially, the input image undergoes the preprocessing stage, in which the Curvelet transform along with directional denoising is used to enhance the image quality. The Curvelet transform captures the smooth edges and the elongated structures, which are very common features in lung structures. The preprocessing of the image is continued with the image resizing and intensity normalization operations. In the next stage a dual-path strategy is used for feature extraction. To capture both semantic and texture-level information, deep features are obtained from a modified ResNet-50 architecture, and texture features are extracted using the local binary pattern method. The standard ResNet-50 architecture is modified to accept single channel inputs and also an attention module is integrated to focus on the most relevant spatial and channel wise regions. The fused representation of deep and LBP features is given as input to a customized classification head to generate the final output. The class imbalance nature of the dataset is handled by the weighted random sampling technique, which is performed in the training phase. A stratified and well balanced version of the Kaggle Chest Ray dataset is used for fine-tuning the model. The steps of the proposed methodology are illustrated in the enhanced ResNet-50 framework, Figure 2.

### 3.1. Dataset

For this study, the Kaggle Chest X-ray Pneumonia Dataset [51] developed by Paul Mooney is employed, consisting of 5856 grayscale chest X-ray images. In the original dataset, the training set had 1341 normal and 3875 pneumonia images, while the validation set was critically under represented with only 8 images per class. A stratified splitting strategy is applied to maintain the original class proportions across all data partitions, thereby improving model generalizability and reducing sampling bias. In this approach, all the images are aggregated according to their labels such as normal and pneumonia from all the sets in the original dataset. The stratified sampling process is performed on the basis of this aggregated set of labeled image paths. A first level stratified split is performed by dividing seventy percent of dataset into the training set and the remaining thirty percent into the temporary set, ensuring both sets contained the same proportion of normal and pneumonia images as the original dataset. A second level stratified split is performed on the temporary set to obtain the final validation set and test set, each containing fifteen percent of the data by preserving the class distribution. As a result of this two level stratification, each subset of the dataset contains a balanced train, test, and validation set, which is represented in Table 2.

Analyzing the stratified dataset details, it is clear that the pneumonia class constitutes about 72% of the samples in each subset of the dataset. To address this issue, a weighted random sampler function is performed during model training. In this method, sampling weights are applied inversely according to their class frequency. So, the class with a lower number of samples will be getting more weights than the classes with a larger number of samples. Unlike oversampling or downsampling techniques, this strategy retains the full dataset and preserves real-world class proportions.

### 3.2. Data Preprocessing

Preprocessing is a vital step in improving the informative content of chest radiographs, facilitating more accurate and effective interpretation. The preprocessing pipeline consists of three major components, such as Curvelet transform with directional denoising, image resizing, and normalization.

#### 3.2.1. Curvelet Transform with Directional Denoising

Medical images contain Gaussian and Poisson noises, which obscure the fine features present in the chest radiograph images. Curvelet transform outperforms the other wavelet transforms in capturing the smooth edges and the elongated structures. Let f(x, y) be the grayscale input image. The Curvelet transform $C_{j,l,k}$ [52] of the image can be expressed as

(1) $$C_{j,l,k}=\langle f\left(x,y\right),\psi_{j,l,k}\left(x,y\right)\rangle$$

where *j*, is the scale index, *l* represents the orientation, *k* represents the spatial position, and $\psi_{j,l,k}$ is the Curvelet basis function.

After decomposition, the noise is removed by using directional filters, such as the Gabor and anisotropic Gaussian filters. Gabor filters [53] are sensitive to orientation and frequency. It gives importance to ridge like pneumonia patterns. On the other hand, anisotropic filters [54] preserve directional structure and random noise.

After applying the directional filters, the inverse Curvelet transform is used for the reconstruction of the denoised image from the coefficients using the equation

(2) $$\hat{f}\left(x,y\right)=\sum_{j,l,k}{\hat{C}}_{j,l,k}\cdot \psi_{j,l,k}\left(x,y\right)$$

where ${\hat{C}}_{j,l,k}$ are the modified coefficients obtained after directional denoising using filter banks.

The weighted averaging of the directionally filtered outputs is used for the final denoised image as follows:

(3) $$I_{\text{MCF-DD}}=\alpha \cdot I_{\mathrm{Gaussian}}+\beta \cdot I_{\mathrm{Gabor}}+\gamma \cdot I_{\mathrm{Anisotropic}}$$

with α+β+γ=1, typically chosen as α=0.4, β=0.3, and γ=0.3.

#### 3.2.2. Image Resizing

For CNN architectures, all input images should be resized to the fixed dimension 224 × 224, which ensures a consistent tensor shape for batch processing and also makes the framework compatible with the pretrained backbone ResNet-50.

#### 3.2.3. Image Normalization

Normalization is used for mapping the input image pixels within a common range. Min-Max normalization is used, which prevents saturation of activation functions and improves the convergence of gradient-based optimization.

### 3.3. Feature Extraction

#### 3.3.1. Extraction of Deep Features

In order to capture the deep features from chest radiograph images, different deep learning architectures were considered. Among these architectures ResNet-50 was selected as the backbone for the extraction of deep features due to its specific advantages over the other models. The vanishing gradient problem observed in deeper networks was resolved in ResNet-50 by its deep architecture, built upon residual learning with identity shortcut connections. Also, ResNet-50 provides a balanced tradeoff between depth and computational efficiency. To capture the deep features from chest radiograph images for pneumonia detection, a modified ResNet-50 architecture is used. The model uses both residual learning and attention mechanisms to focus more on infection-affected lung regions while suppressing irrelevant background structures.

##### Input Adaptation

The standard ResNet-50 architecture [55,56] is designed to accept three channel RGB images, on the other hand, chest radiographs are grayscale images. To adapt the ResNet-50 model to accommodate this change, the first convolutional layers are modified to receive and process one channel instead of three channels. The first convolutional layer extracts fundamental structural elements like edges and corners from the input image. Sixty four filters with a kernel size of 7 × 7 are used in this layer. This large kernel size captures the board spatial features.

##### Max Pooling Layer

To reduce spatial resolution and control overfitting, a Max Pooling layer with a stride of 2 and kernel size of 2 × 2 is employed, downsampling the feature maps from 112 × 112 to 56 × 56, in which a kernel size of 3 × 3 is used. This stage introduces local translation invariance, which helps the model to remain robust to small shifts in the feature location. Max Pooling ensures that the important visual signals are dominant, preventing irrelevant background textures.

##### ResNet-50 Architecture

The core part of the ResNet-50 architecture is the four stages of the bottleneck residual block, which solves the vanishing gradient problem by using shortcut connections. There are three convolutional layers for each bottleneck layer, 1 × 1 for compression, 3 × 3 for spatial processing, and 1 × 1 for expansion, which is followed by a shortcut connection that implements residual learning. The feature channel is increased from 64 to 2048 as a result of this residual architecture, which is given in Table 3.

At the end of the ResNet-50 architecture, the deep network provides outputs of a 2048-dimensional feature vector for each input image. Each of these 2048 values produces a different learned pattern, such as edges, lung opacity patterns, etc. These are known as deep features, and they are rich, high-level representations of the image content.

##### Convolutional Block Attention Module

After completing the final convolutional operations, CBAM [57] is embedded into the architecture to introduce spatial and channel-wise attention. This module analyzes the interchannel relationships in the input $F\in R^{C\times H\times W}$ 2048 and generates a channel attention map $M_{c}\in R^{C\times 1\times 1}$ that weights each channel according to their significance. For the computation of $M_{c}$, the two descriptors used are Global Average Pooling and Global Max Pooling, which can be mathematically represented as

(4) $$f_{\mathrm{avg}}^{c}=\frac{1}{H\times W}\sum_{i=1}^{H}\sum_{j=1}^{W}F\left(c,i,j\right)$$

(5) $$f_{\max}^{c}=\max_{i,j}F\left(c,i,j\right)$$

These descriptors are passed through Perceptron with multiple layers, which contains layers such as a hidden layer and ReLU activation layer. The outputs are summed and passed through a sigmoid activation function:

(6) $$M_{c}\left(F\right)=\sigma \left(\mathrm{MLP}\left(f_{\mathrm{avg}}^{c}\right)+\mathrm{MLP}\left(f_{\max}^{c}\right)\right)$$

The final channel attention map is applied to the input feature map using element-wise multiplication:

(7) $$F^{\prime }=M_{c}\left(F\right)⊗F$$

##### Global Averaging Layer

The attention-refined feature map $F^{\prime \prime }\in R^{C\times H\times W}$ from the CBAM layer is passed to a Global Average Pooling (GAP) operation. The GAP layer then compresses the spatial dimensions, generating a compact and discriminative feature vector. The GAP operation is defined as

(8) $$f_{c}=\frac{1}{H\times W}\sum_{i=1}^{H}\sum_{j=1}^{W}F^{\prime \prime }\left(c,i,j\right),\;\mathrm{for}\;c=1,2,\ldots ,C$$

The resulting output is a *C*-dimensional vector:

(9) $$f=\left[f_{1},f_{2},\ldots ,f_{C}\right]\in R^{C}$$

This feature vector refined through both attention and spatial aggregation fuses together the texture descriptor features in the next stage.

### 3.4. Texture Feature Extraction from Original and LBP Images

A set of texture features were extracted from both the original chest X-ray image and its local binary pattern (LBP) transformed image. The original images have rich gray level intensity variations and structural anatomy and texture patterns that represent the global and local lung tissue characteristics. But the small and granular patterns will be missed when processing the raw input image. So, to capture the micropatterns, LBP transformation is applied where the local texture variations are encoded as relationship between a pixel and its neighbors. By extracting features from both, we will be able to extract the global structure features and local texture pattern features.

#### 3.4.1. Local Binary Pattern (LBP) Transformation

Local binary pattern [58] is a powerful feature descriptor for a texture classification. For a given pixel $I_{c}$, the local binary pattern (LBP) is computed by thresholding its *P* neighbors using a circular radius *R*:

(10) $${\mathrm{LBP}}_{P,R}=\sum_{p=0}^{P-1}s\left(I_{p}-I_{c}\right)\cdot 2^{p}$$

where the function s(x) is defined as

(11) $$s\left(x\right)=\left\{\begin{array}{cc} 1 & \mathrm{if}\;x\geq 0\\ 0 & \mathrm{otherwise} \end{array}\right.$$

A uniform LBP operator with P = 8 and R = 1 is used in the framework, which produces a 26-bin histogram representing uniform patterns. The resulting LBP image highlights local edge, spot, and flat region structures.

#### 3.4.2. Texture Descriptors from Original and LBP Images

The following texture features are extracted from both the original chest radiograph and its corresponding LBP images.

##### Gray-Level Co-Occurrence Matrix (GLCM)

The Gray-Level Co-occurrence Matrix (GLCM) [59,60] is used to analyze texture by measuring the spatial relationships between pairs of pixels at defined orientations and distances within the image. From the GLCM matrix, the following statistical features such as contrast, Correlation, Energy, and Homogeneity are calculated. Each of these four features are calculated for four values of

$$\theta \in \{0^{\circ },45^{\circ },90^{\circ },135^{\circ }\}$$

and so a total of 16 GLCM features are extracted for a single image.

##### Shannon Entropy

Shannon Entropy [61] is a statistical measurement which gives the randomness in the intensity distribution. It quantifies the disorder in the texture pattern. A higher entropy value represents a higher degree of disorder in the image intensities, which can occur as a result of some abnormalities in the image. It can be defined as

(12) $$H=-\sum_{i=1}^{N}p_{i}\log_{2}\left(p_{i}\right)$$

where $p_{i}$ is the probability of occurrence of the *i*th gray level in the image histogram, and *N* is the number of gray levels. A higher entropy value indicates a higher degree of texture complexity or disorder.

##### Hu’s Invariant Moments

The invariant in the image patterns are represented by Hu’s Invariant moments [62]. These are a set of seven features derived from central image moments that are invariant to translation, scale, and rotation. The normalized central moments $\eta_{pq}$ are given by

(13) $$\eta_{pq}=\frac{\mu_{pq}}{\mu_{00}^{\left(1+\frac{p+q}{2}\right)}}$$

##### Zernike Moments

To calculate the Zernike moments, image pixels are projected onto orthogonal polynomials defined over the unit disk. Zernike polynomials form a set of orthogonal basis functions on the unit disk, allowing for compact representation of the shape information. A total of 10 Zernike moments are extracted per image, comprising 5 magnitudes and 5 phase components to capture rotation-invariant global shape features from both the original and LBP-transformed chest X-ray images [63].

##### Fractal Dimension (FD)

The complexity and self-similarity of texture patterns within an image are quantified by this feature. One of the most commonly used approaches to estimate the FD is the box-counting method, which evaluates how detail in a pattern changes with scale.

##### LBP Histogram Statistics

From the 26-bin uniform LBP histogram, we compute 5 statistical descriptors, such as the mean, standard deviations, skewness, kurtosis, and histogram entropy. These features provide a summarized representation of local binary patterns. These features are extracted from the LBP image only.

#### 3.4.3. Feature Fusion Layer

In the feature fusion layer, the deep features from the modified ResNet-50 architecture and the texture descriptor features from the original and its LBP image are fused together. Early fusion, known as feature-level fusion, is used in this framework where the deep features are concatenated with the texture feature by the direct concatenation method. In this method the features are combined into a single vector by joining them end to end, without applying any transformation or weighting beforehand. As shown in Table 4, the expanded summary of the fused feature vector illustrates the distribution and characteristics of the combined LBP and deep features used for classification.

With direct concatenation, you form a new vector:

(14) f=[d∥h]

where

- $d\in R^{2048}$ is the deep feature vector;
- $h\in R^{75}$ is the handcrafted feature vector;
- $f\in R^{2123}$ is the resulting fused feature vector.

**Table 4:** Expanded summary of the fused feature vector.

Feature Type	Source	Feature Count
Deep Features (ResNet-50)	Chest X-ray Image	2048
GLCM Features (0°, 45°, 90°, 135°)	Original + LBP Image	16×2=32
Shannon Entropy	Original + LBP Image	1×2=2
Hu’s Invariant Moments	Original + LBP Image	7×2=14
Zernike Moments (Mag. + Phase)	Original + LBP Image	10×2=20
Fractal Dimension	Original + LBP Image	1×2=2
LBP Histogram Statistics (mean, std, skew, kurtosis, entropy)	LBP Image Only	5
Total Handcrafted Features	—	75
Total Fused Feature Vector	—	2048 + 75 = 2123

##### Feature Normalization

It is essential to normalize the combined feature vector because the features originate from different domains and will have their values in different numerical ranges. Deep features may span large continuous ranges, while handcrafted texture descriptors like GLCM contrast or Hu’s moments often lie within much smaller intervals. Without normalization, high magnitude features can dominate the learning process, causing the classifier to ignore informative but smaller-scaled features. To ensure the equal contribution of all features and to improve training stability and convergence, feature normalization becomes a critical step. Min Max normalization is used, which rescales each feature dimension to lie within the range of [0, 1].

##### Feature Selection

After feature fusion and normalization, the resultant feature vector contains 2123 features for a single image. This feature vector may contain redundant, irrelevant features. Processing these types of features causes problems, like increased computational cost and reduced model generalization capability. Recursive Feature Elimination (RFE) [64] with a linear Support Vector Machine (SVM) as the base estimator is used as the feature selection strategy. RFE works by recursively training the model, ranking features based on their importance. This ranking continues until the optimal number of features is retained, determined through cross-validation performance on the validation set.

The number of features to be selected was not fixed in the initial stage. After applying Recursive Feature Elimination (RFE) with a linear Support Vector Machine, each feature in the fused vector was ranked according to its importance score. The importance of each feature in an SVM model can be inferred from the absolute magnitude of its corresponding weight in the learned decision function, with higher magnitudes suggesting stronger influence on the classification outcome. The value of the weights ranges from 0 to 1, where 1 represents strong features and values closer to 0 represent weaker features. We evaluated subsets of features based on these scores, specifically the top 500, 1000, and 1500. Among them, the top 1000 features, identified based on Recursive Feature Elimination (RFE) scoring, yielded the highest classification performance in terms of accuracy, AUC, and F1-score. Consequently, these top 1000 features were selected for the final model. Out of 1000 features, 860 features are deep features and 140 are texture descriptor features. The top 15 features, selected using Recursive Feature Elimination (RFE) with a linear Support Vector Machine (SVM) classifier, are presented in Table 5.

#### 3.4.4. Softmax Classification

The selected features are given as input to the Softmax classifier [65], which performs the final classification. The classifier was trained using cross-entropy loss to optimize class separation. Since this is a binary classification task of classifying the image to pneumonia or normal, the output corresponds to the probability of each class, and the final prediction is made by choosing the class with the maximum probability. This approach ensures interpretability, probabilistic confidence, and compatibility with modern neural and statistical modeling frameworks. After training, the Softmax classifier outputs class probabilities for each sample.

## 4. Results

### 4.1. Evaluation Metrics

The evaluation metrics used for analyzing the performance of the proposed pneumonia detection framework are accuracy, specificity, sensitivity, precision, and area under the curve.

- Accuracy (%): It is calculated by taking the ration of correctly classified instances over the total number of cases.
- Sensitivity (%): This metric indicates the model’s ability to correctly identify pneumonia positive cases.
- Specificity (%): This model is used to represent the model’s ability to correctly identify pneumonia negative cases.
- Precision (%): The proportion of correctly identified pneumonia cases among all predicted pneumonia cases is given by this metric.
- Area Under the Curve (AUC): This represents the model’s ability to discriminate between the pneumonia and normal classes across all thresholds. An AUC of 1.0 indicates perfect classification, while 0.5 represents random chance.

#### Quantitative Evaluation

For the experimental evaluation, the publicly available chest ray dataset is used. The stratified dataset is used where 70% is used for training and 15% is used for testing and validation each. But as we can see in Table 2, in the stratified dataset, the number of pneumonia cases is 72% of the samples in each subset. To handle this class imbalance problem, the weighted random sampler function is used and a comparative study between two training settings is performed. In the first variant, standard uniform sampling and unweighted loss is implemented, and in the second one, an integrated approach combining weighted random sampling, class-weighted loss, and targeted augmentation is used. The baseline ResNet model without imbalance handling exhibited high overall accuracy of 83.6% but significantly lower sensitivity of 93.2% for the minority normal class, which indicates a strong bias toward the majority pneumonia class. After applying class imbalance techniques, the updated model achieved an improved accuracy of 85.9%, with sensitivity increasing to 98.2%.

For the quantitative evaluation of the improved ResNet-50 architecture, four different models were evaluated.

- Variant 1: Standard ResNet-50.
- Variant 2: ResNet-50 with CBAM.
- Variant 3: ResNet-50 with CBAM and LBP Fusion.
- Variant 4: ResNet-50 with MCF-DD and CBAM and LBP Fusion.

To systematically evaluate the effects of preprocessing, feature fusion, and attention mechanisms on classification performance, four ResNet-50-based model variants were implemented. The baseline model utilized the standard ResNet-50 architecture with class imbalance handling mechanisms.

A second variant was considered in which a Convolutional Block Attention Module (CBAM) was integrated after the final convolutional block of ResNet-50 to enhance the model’s ability to focus on pathology relevant regions by applying both spatial and channel attention. In the third variant, ResNet-50 with the CBAM module was considered. In this model, the deep features from ResNet-50 were fused with local binary pattern (LBP) descriptors extracted from the original X-ray images and the LBP images. These combined features were concatenated before the final dense layers. Recursive Feature Elimination (RFE) was used to select the top 1000 most informative features, which were then normalized and forwarded to the classification head. The fourth variant incorporated a preprocessing step using Multi Curvelet Filtering with Directional Denoising (MCF-DD), applied to the input X-rays prior to feature extraction. This preprocessing pipeline included Curvelet based decomposition followed by directional filtering and adaptive coefficient fusion, aiming to suppress noise and enhance pathological structures. The fused features from ResNet-50 and LBP were then passed through CBAM-enhanced blocks to emphasize diagnostic regions before classification.

All the models were trained using a categorical cross-entropy loss function with the Adam optimizer with an initial learning rate = 1 × 10^−4^ and a batch size = 32 for 50 epochs. Table 6 presents the performance comparison of the four model variants, highlighting the differences in accuracy, sensitivity, specificity, precision and AUC, in which the best performing results in each column are highlighted in bold.

**Figure 3:**
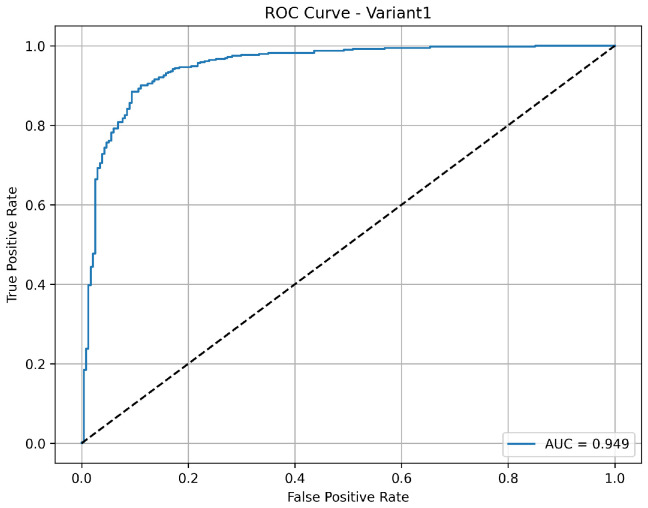
ROC curve for variant 1 showing AUC performance.

A balanced and clinically meaningful performance is achieved by variant 1 with a classification accuracy of 85.9%. Sensitivity with 98.2% indicates a strong capability to identify the pneumonia cases correctly. But the specificity is relatively lower at 65.4%, suggesting a higher rate of false positives for normal cases. The precision of 82.5% reflects good confidence in positive and negative predictions, respectively. With an AUC of 0.949 as shown in Figure 3, the model demonstrates robust discriminative ability between the classes, confirming its effectiveness, especially in identifying diseased cases with high recall.

**Figure 4:**
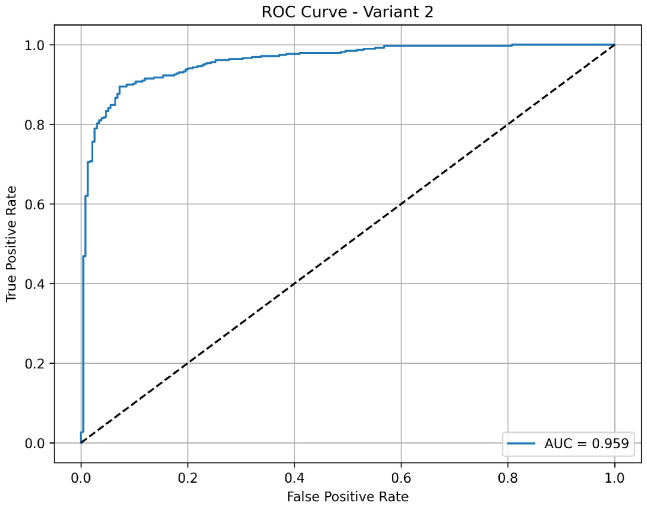
ROC curve for variant 2 showing AUC performance.

Variant 2 demonstrates a strong pneumonia detection capability with a very high sensitivity of 97.2%, meaning it effectively identified nearly all pneumonia cases. However, its specificity is relatively low at 57.7%, indicating more false positives among normal cases, which reduces its overall accuracy to 82.4%. The precision of 79.3% suggests that while positive predictions include some false alarms, the negative predictions are still highly reliable. The AUC of 0.960 as shown in Figure 4 confirms that the model maintains good class separability, but the lower specificity may impact clinical usability where reducing unnecessary alerts is critical.

**Figure 5:**
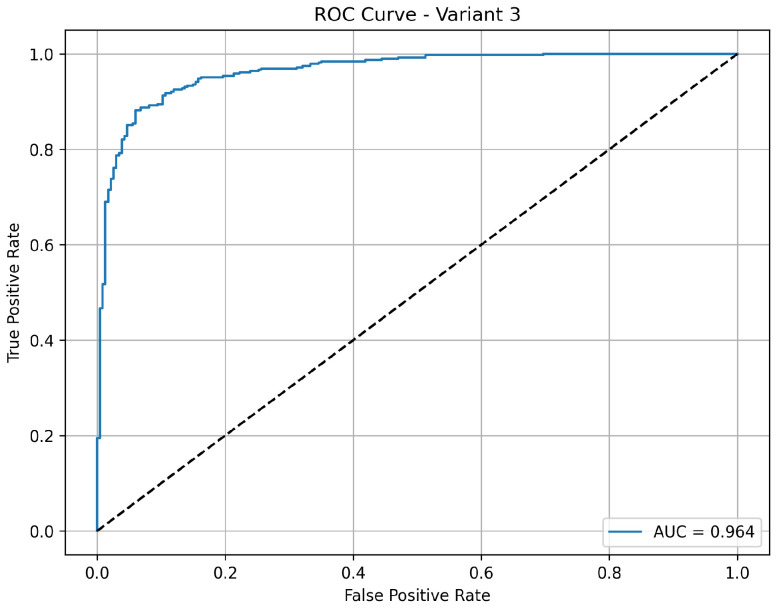
ROC curve for variant 3 showing AUC performance.

Variant 3 achieves a strong balance between sensitivity and overall classification performance. It reports an accuracy of 84.6%, with a notably high sensitivity of 98.5%, indicating its consistent ability to correctly detect pneumonia cases. The specificity is 61.5%, showing a moderate improvement in correctly identifying normal cases compared to variant 2. The precision of 81% indicates reliable positive predictions. With an AUC of 0.963 as shown in Figure 5, this variant shows excellent discriminative power between normal and pneumonia cases, making it a well-rounded model for clinical applications.

**Figure 6:**
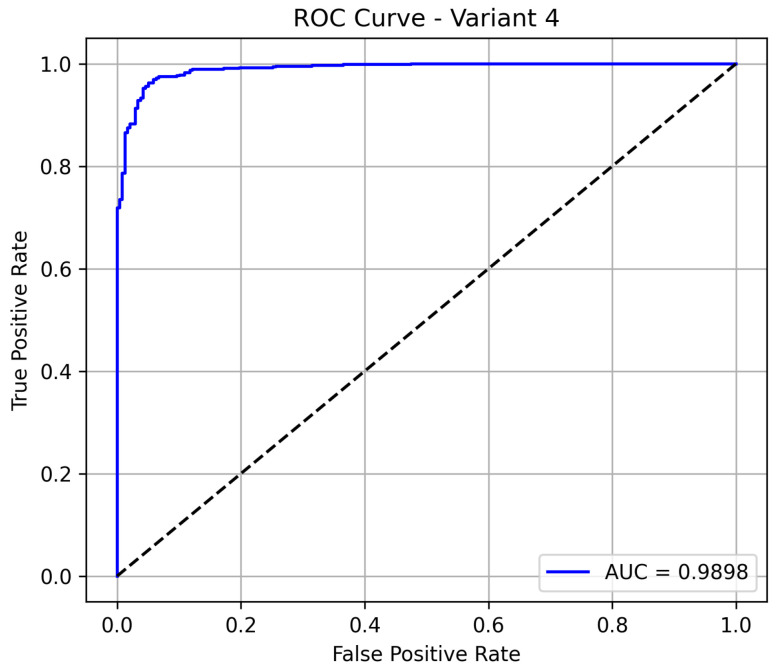
ROC curve for variant 4 showing AUC performance.

Variant 4 delivers the best overall performance among all the evaluated models, achieving an accuracy of 96.08%, which indicates a highly reliable classification system. It maintains a sensitivity of 95.95%, confirming its strong ability to detect pneumonia cases, while also significantly improving its specificity to 94.6%, minimizing false positives in normal cases. The model demonstrates excellent precision at 98.09%. Notably, the model achieves an AUC of 0.985 as shown in Figure 6, demonstrating excellent discrimination between pneumonia and normal cases. This positions variant 4 as the most reliable and clinically effective configuration.

**Figure 7:**
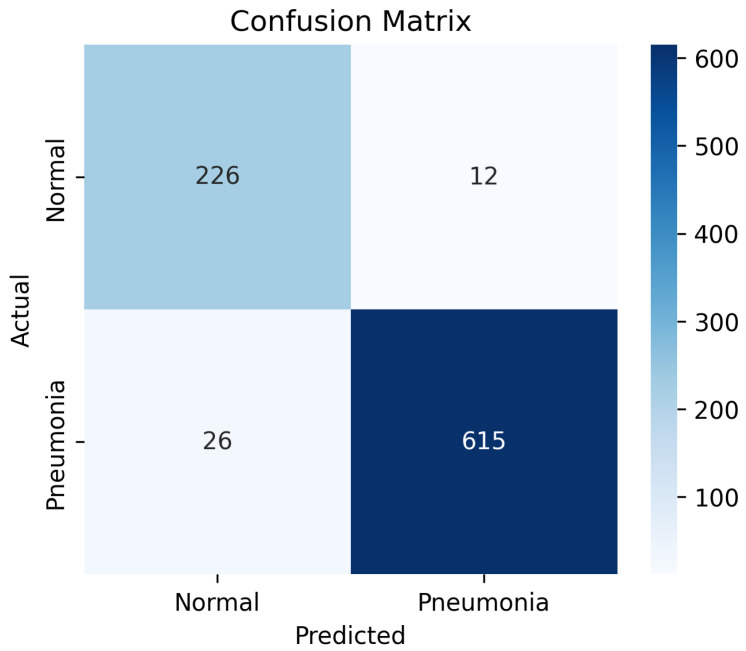
Confusion matrix for Variant 4.

The confusion matrix in Figure 7 demonstrates the classification performance of the proposed model on the test dataset. Out of the total 238 normal cases, 226 were correctly classified as normal, while 12 were misclassified as pneumonia, indicating a relatively low false-positive rate. Similarly, among 641 pneumonia cases, 615 were accurately identified, with only 26 instances misclassified as normal, reflecting a low false-negative rate. These results correspond to a high overall classification accuracy and balanced sensitivity and specificity.

### 4.2. Qualitative Assessment of True and False Predictions Using Grad-CAM

**Figure 8:**
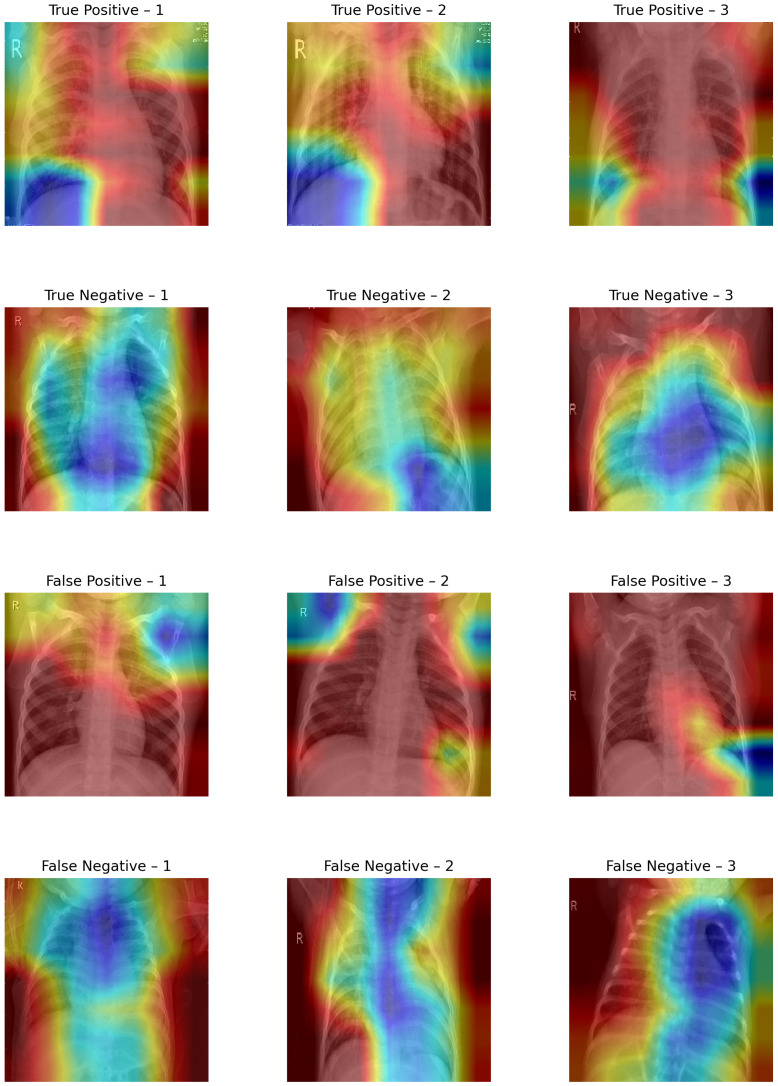
Grad-CAM visualization.

The Grad CAM visualizations shown in Figure 8 provide a qualitative interpretation of the model’s decision-making process across different prediction outcomes.

In true-positive (TP) instances (615 samples), the Grad-CAM maps predominantly highlight pneumonia-affected regions, with dense activations over lung fields, especially the lower lobes, suggesting that the model has successfully learned to localize pathological features. This strong spatial correspondence justifies the high sensitivity observed in the evaluation.

In true-negative (TN) cases (226 samples), the activation maps display scattered or minimal intensity across noncritical lung regions, affirming that the model avoids overattending to normal anatomical structures. The background-focused attention confirms that no abnormal features influenced the model’s decision, supporting the 95% specificity.

Conversely, in false-positive (FP) samples (12 instances), the visualizations often show misdirected attention toward ribs, cardiac shadows, or peripheral noise, indicating that subtle structural textures have been misinterpreted as pneumonia markers. This highlights the model’s vulnerability to non-pathological variations and borderline cases.

False-negative (FN) visualizations (26 samples) typically reveal weak or misplaced activations, often missing the true infection zones, suggesting that the model failed to recognize subtle or atypical pneumonia presentations. These errors underline the challenge of detecting early-stage or mild infections where visual cues may be ambiguous.

Across the evaluated configurations, variant 4 proved to be the most effective model, attaining the highest classification accuracy of 96.08%, specificity of 94.96%, and AUC of 0.98 while maintaining an excellent sensitivity of 95.95%. This indicates that variant 4 not only detects pneumonia cases effectively but also significantly reduces false positives, making it highly reliable in clinical settings.

## 5. Discussion

The proposed system uses a hybrid approach by combining the deep features extracted from a modified ResNet-50 with handcrafted texture features derived from both original and LBP transformed chest X-ray images. This integration captures both high level semantic representations and low level structural patterns, improving classification performance, especially in distinguishing subtle textural differences between pneumonia and normal lungs.

A high sensitivity of 95.95% ensures that most of the pneumonia cases are identified accurately. This is crucial in clinical screening applications, where minimizing false negatives is essential to avoid missed diagnoses. The fusion of features enhances representational richness, and the inclusion of handcrafted descriptors like GLCM, entropy, Hu moments, and Zernike moments allow for improved interpretability and robustness. Furthermore, variant 4 achieved a balanced improvement in both sensitivity and specificity, indicating the strength of feature normalization and selection in reducing overfitting.

### Computational Efficiency and Practicality

To assess the computational efficiency of the proposed RFE-based feature selection strategy, we benchmarked its training and inference times against the baseline ResNet-50 model. Feature extraction using the pretrained ResNet-50 took approximately 1.8 min. The subsequent Recursive Feature Elimination (RFE) step, applied to reduce the original 2048 features to the top 1000 most informative features using a linear SVM estimator, required about 2.0 min. The total training time for the proposed pipeline was around 3.8 min. During inference, the reduced feature set enabled faster classification, with an average prediction time of 60 ms per image, compared to 90 ms in the baseline ResNet-50 model without feature reduction. These experiments were performed on Google Colab Pro utilizing an NVIDIA A100 GPU (NVIDIA Corporation, Santa Clara, CA, USA). The results indicate that the proposed feature selection method significantly improves classification performance while also reducing inference latency, rendering it highly suitable for time-sensitive clinical applications. Computational cost and performance comparison between baseline ResNet-50 and proposed framework in given in the Table 7.

Despite its strong performance, the system exhibits certain limitations. Variants 1 to 3 showed lower specificity, indicating a higher false-positive rate, which could result in over-diagnosis when used as a standalone tool. The high dimensionality of features prior to selection increases training time and memory usage. Moreover, the reliance on LBP transformed images assumes that texture information is consistently diagnostically relevant, which may not hold across all imaging conditions or datasets. Furthermore, the current framework is limited to 2D chest radiographs, does not scale to volumetric data such as 3D CT scans, and lacks clinical validation through expert radiologist. Comparison of recent studies for pneumonia detection using chest X-rays Kaggle dataset with the proposed framework is depicted in Table 8.

Future research can address these challenges through several directions. Extending the method to multiclass classification, such as differentiating between bacterial and viral pneumonia or other chest pathologies, will improve its clinical applicability. Finally, validating the system on larger, diverse, and real world datasets will ensure robustness and generalization. In future work, we plan to conduct a radiologist in the loop evaluation to further assess the clinical relevance and diagnostic reliability of the proposed framework. This will involve comparing the model’s predictions with annotations and diagnoses provided by multiple expert radiologists.

## 6. Conclusions

A hybrid structured framework for detecting pneumonia from chest X-ray images by combining deep features extracted from ResNet-50 with handcrafted texture descriptors from both original and LBP-transformed images is discussed in this work. Feature fusion was performed through direct concatenation, followed by normalization and Recursive Feature Elimination to reduce dimensionality and enhance discriminative capability. Among the evaluated variants, the proposed variant 4 achieved the highest performance, demonstrating the benefit of integrating handcrafted features with deep representations. This hybrid approach not only enhances classification accuracy but also plays a significant role in improving the interpretability and robustness, making it a promising tool for supporting computer-aided diagnosis in practical, real-world clinical environments.

## Figures and Tables

**Table 1:** Recent studies on pneumonia detection from chest X-ray images.

S.No	Title	Methodology	Dataset	Contribution	Challenges
1	Interpretable DL for Pneumonia [37]	ResNet-50 and CAM and LRP	Kaggle Chest X-ray Images	Robust classification with explainability	Lack of interpretability
2	MobileNet-based Pneumonia Detection [38]	MobileNetV2	Kaggle Chest X-ray Images and ChestX-ray14	Lightweight model suitable for mobile deployment	Lack of explainability tools and dataset bias
3	Ensemble DCNNs for Pneumonia Detection [39]	DenseNet169, MobileNetV2, and Vision Transformer	Kaggle Chest X-ray Image	Fusion of architectures for robustness	Hyper parameter tuning and lack of interpretability
4	Pneumonia Detection with Weak Labels [40]	Weak supervision with ResNet34	Kaggle Chest X-ray Images and RAIG dataset	Accurate detection and localization with weak supervision	Lack of quantitative analysis for localization accuracy
5	Hybrid Inception-Residual Model [41]	Fine-tuned Inception ResNetV2 and transfer learning	Labeled Optical Coherence Tomography (OCT) and Chest X-Ray Images	Superior feature extraction and computational efficiency	Model generalization and overfitting
6	Pneumonia Detection via CNN-ViT Fusion [42]	ResNet34 and Vision Transformer fusion	Kaggle Chest X-ray Images	Combined local/global attention for classification	Image quality requirements
7	Cardio-Respiratory Disease Detection via DL Ensembles [43]	Deep Learning Ensemble approach	NIH Chest X-ray dataset	Architecture fusion for reliability	Lack of model interpretability and computational complexity
8	Radiologist-Level DL (CheXNet) [44]	DenseNet-121 pretrained	ChestX-ray14	Exceeded radiologist-level performance	Computational complexity
9	AI Model for Pneumonia Classification [45]	Ensemble classifiers	NIH ChestX-ray14 Dataset	Hybrid AI framework combining deep features and PSO-based feature optimization	Absence of explainability
10	DL-Based Lung Condition Classifier (CXR + CT) [46]	Transfer learning model	Kaggle dataset	Sorting system for COVID-19 implemented	Higher computational resources and time
11	ViT-Based Pneumonia Detection [47]	Vision Transformer (ViT) architecture	Kaggle Chest X-ray (CXR)	Proposal of a ViT-based Architecture	Data scarcity and real world validation
12	Transformer Model for X-ray Pneumonia Detection [48]	Ensembling Vision Transformer and Convolutional Neural Network	Kaggle Pediatric Pneumonia Dataset	Improved accuracy using ViT	High computational resource requirement
13	TB Classification via CNN and Transformer [49]	Ensembling data-efficient image transformer and the Residual Network-16 model	TBX11K dataset	Hybrid CNN-Transformer detection model	Limited generalization due to dataset bias
14	Twin-layer attention graph convolutional network (TLA-GCN): Enhancing abnormality detection in chest X-rays [50]	Twin-Layer Attention Graph Convolutional Network (TLA-GCN)	NIH Chest X-ray and COVID-19 dataset	Better representation of inter-region dependencies in chest X-rays	High computational cost

**Table 2:** Stratified dataset details.

Subset	Normal Images	Pneumonia Images	Total Images
Train	1108	2991	4099
Test	237	641	878
Validation	238	641	879

**Table 3:** ResNet-50 architecture-residual block.

Stage	Layer Name	Output Size
1	Convolution layer 1	112×112×64
2	MaxPool	56×56×64
3	Convolution layer 2	56×56×256
4	Convolution layer 3	28×28×512
5	Convolution layer 4	14×14×1024
6	Convolution layer 5	7×7×2048

**Table 5:** Top 15 features selected using Recursive Feature Elimination (RFE) with linear SVM.

Rank	Feature Name	Type	Score
1	Deep_Feature_1493	Deep (ResNet-50)	1.000
2	GLCM_Contrast_90deg_LBP	Handcrafted	0.986
3	Deep_Feature_876	Deep (ResNet-50)	0.974
4	Zernike_Magnitude_3_org	Handcrafted	0.966
5	Deep_Feature_112	Deep (ResNet-50)	0.954
6	Hu_Moment_2_LBP	Handcrafted	0.942
7	Deep_Feature_1985	Deep (ResNet-50)	0.939
8	FD_LBP	Handcrafted	0.932
9	Deep_Feature_320	Deep (ResNet-50)	0.921
10	LBP_Histogram_Entropy	Handcrafted	0.915
11	Deep_Feature_2021	Deep (ResNet-50)	0.901
12	Zernike_Phase_2_LBP	Handcrafted	0.899
13	Deep_Feature_1055	Deep (ResNet-50)	0.885
14	Shannon_Entropy_Orig	Handcrafted	0.874
15	GLCM_Homogeneity_135deg_org	Handcrafted	0.861

**Table 6:** Performance comparison of four model variants.

Metric	Variant 1	Variant 2	Variant 3	Variant 4
Accuracy (%)	85.90	82.40	84.60	**96.08**
Sensitivity	0.982	0.972	**0.985**	0.9595
Specificity	0.654	0.577	0.615	**0.9496**
Precision (PPV)	0.825	0.793	0.810	**0.9809**
AUC	0.949	0.960	0.963	**0.985**

**Table 7:** Computational cost and performance comparison between baseline ResNet-50 and proposed framework.

Metric/Step	Baseline ResNet-50	Proposed RFE- Optimized Pipeline
Feature Extraction Time	1.8 min	1.8 min
Feature Selection Time (RFE-1000 feats)	—	2.0 min
Total Training Time	1.8 min	3.8 min
Inference Time per Image	90 ms	60 ms
Classification Accuracy	85.90%	96.02%

**Table 8:** Comparison of recent studies for pneumonia detection using chest X-rays.

Study	Dataset	Accuracy (%)	Sensitivity (%)	Specificity (%)	AUC/F1 Score
Pneumonia Classification via MobileNet [38]	Kaggle Chest X-Ray Images [51]	94.23	–	–	-
DCNN with Attention Ensemble for Pneumonia [66]	Kaggle Chest X-Ray Images [51]	95.19	93.84	97.43	AUC: 0.9564
CNN-Based Pneumonia Detection with Integrated Gradients [67]	Kaggle Chest X-Ray Images [51]	97.23	–	–	–
Model-Level Ensemble of CNN and ViT for Pneumonia Detection [68]	Kaggle Chest X-Ray Images [51]	94.87	–	–	–
Proposed-Enhanced ResNet-50 (Variant 4 )	Kaggle Chest X-Ray Images [51]	96.08	95.95	94.96	AUC: 0.98

## Data Availability

The chest X-ray images used in this study are part of the publicly available Chest X-Ray Images (Pneumonia) dataset, provided by Guangzhou Women and Children’s Medical Center, China, and hosted on Kaggle [51]. The dataset is fully anonymized, containing no personally identifiable information, and is available under the terms specified on the Kaggle platform.

## Abbreviations

The following abbreviations are used in this manuscript:

CNN	Convolutional Neural Network
CBAM	Convolutional Block Attention Module
LBP	Local Binary Pattern Image
GAP	Global Average Pooling
GLCM	Gray-Level Co-occurrence Matrix
ResNet	Residual Network
